# Information Systems to Support Surveillance for Malaria Elimination

**DOI:** 10.4269/ajtmh.14-0257

**Published:** 2015-07-08

**Authors:** Colin Ohrt, Kathryn W. Roberts, Hugh J. W. Sturrock, Jennifer Wegbreit, Bruce Y. Lee, Roly D. Gosling

**Affiliations:** Malaria Elimination Initiative, Global Health Group, University of California, San Francisco, California; Public Health Computational and Operational Research (PHICOR), Johns Hopkins School of Public Health, Baltimore, Maryland

## Abstract

Robust and responsive surveillance systems are critical for malaria elimination. The ideal information system that supports malaria elimination includes: rapid and complete case reporting, incorporation of related data, such as census or health survey information, central data storage and management, automated and expert data analysis, and customized outputs and feedback that lead to timely and targeted responses. Spatial information enhances such a system, ensuring cases are tracked and mapped over time. Data sharing and coordination across borders are vital and new technologies can improve data speed, accuracy, and quality. Parts of this ideal information system exist and are in use, but have yet to be linked together coherently. Malaria elimination programs should support the implementation and refinement of information systems to support surveillance and response and ensure political and financial commitment to maintain the systems and the human resources needed to run them. National malaria programs should strive to improve the access and utility of these information systems and establish cross-border data sharing mechanisms through the use of standard indicators for malaria surveillance. Ultimately, investment in the information technologies that support a timely and targeted surveillance and response system is essential for malaria elimination.

## Introduction

**Figure 1.**
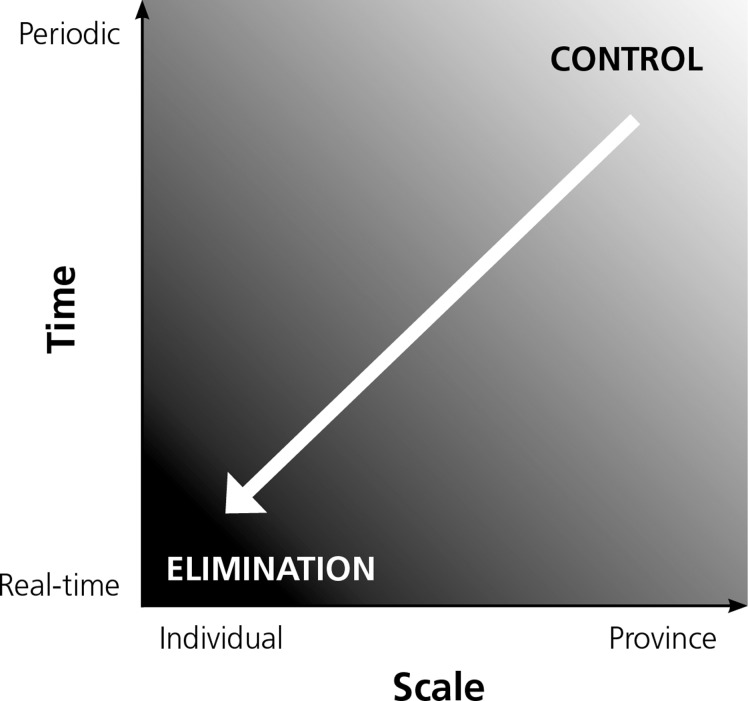
Changes in the spatial and temporal scale of malaria surveillance and response in the shift to elimination (Modified from Cao and others^6^).

Robust and responsive information systems are critical for successful malaria control and elimination.^1^–^5^ In elimination settings, surveillance must be an intervention where data collection, analysis, output, and response occur quickly to identify symptomatic and asymptomatic cases, prevent onward transmission, and reduce vectorial capacity. The best way to ensure that this occurs rapidly and efficiently is to work with information systems designed to support malaria surveillance and response. Specifically, in an elimination setting, case reporting needs to shift from being periodic and aggregated at the district or provincial level to real-time reporting of individual geo-located cases (Figure 1).

Technologies that support elimination surveillance can facilitate many essential elements such as real-time or rapid reporting and case and intervention mapping. Here we describe the characteristics of an ideal malaria elimination information system that has the capacity to identify individual cases, analyze and share information, and stimulate real-time action to prevent onward transmission.

This article is one in a series of four that is intended to guide malaria elimination program decision making. These articles draw on both published and unpublished literature and qualitative data gathered from key informant interviews. This article offers specific recommendations to guide the choice of information systems in elimination settings.

## Methods

These findings were informed by published and grey literature. In addition to a review of publications specific to malaria elimination and other disease eradication, the literature search included combinations of the following topics and search terms: chagas, cross-border data sharing, data management, dengue, geospatial, guidelines, influenza, information systems for health, polio, imported malaria, integrated disease surveillance, inter-country collaboration, malaria elimination, monitoring and evaluation, outbreak alert, rapid reporting, sentinel surveillance, surveillance, World health Organization (WHO), yellow fever, zero reporting, and specific country programs mentioned by key informants. A total of 157 documents were identified and reviewed. The authors conducted 21 key informant interviews with malaria field experts, surveillance specialists, geographic information systems (GIS) experts, information technology experts, and members of malaria control and elimination programs, as well as experts in the control and eradication of diseases other than malaria.

## Characteristics of an ideal elimination information system

**Figure 2.**
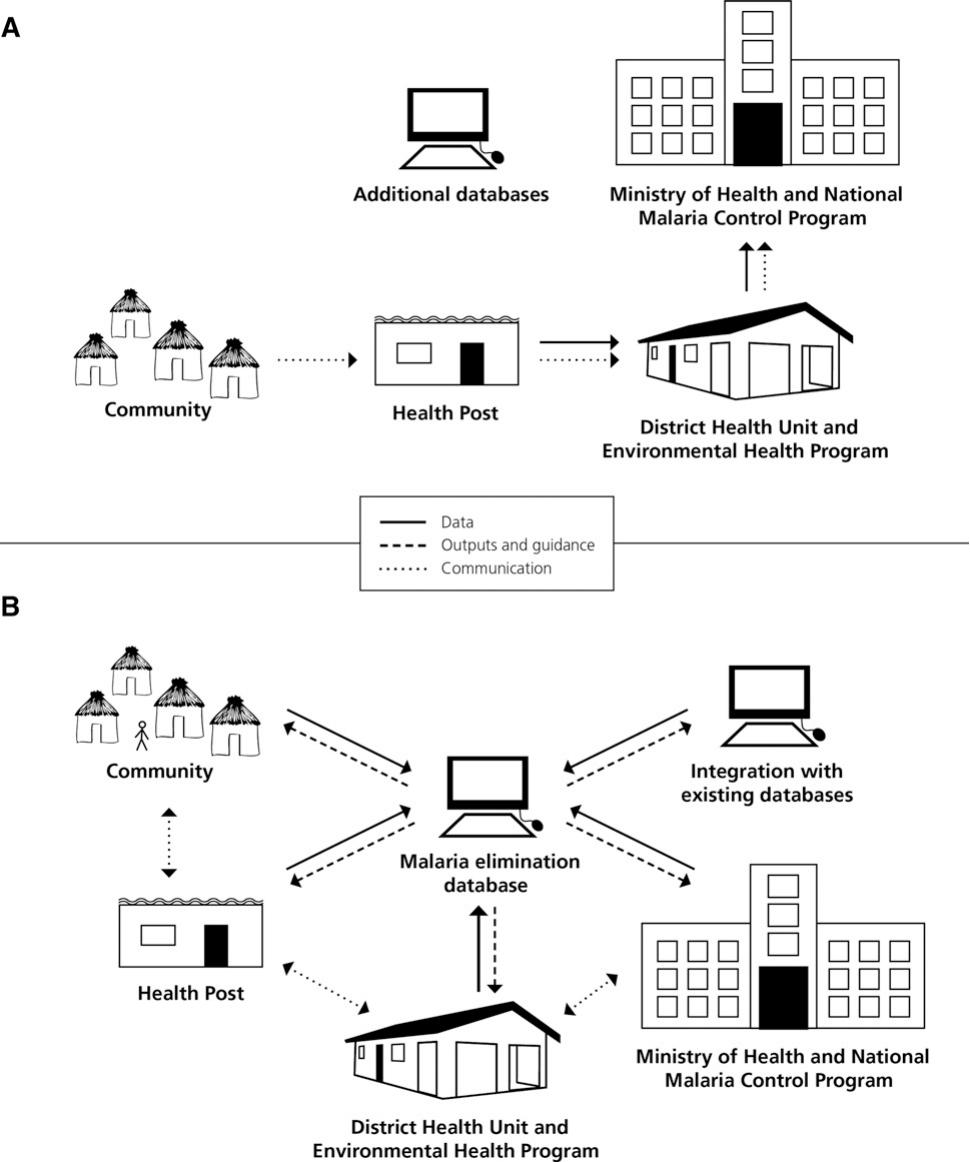
Malaria surveillance systems. (**A**) Traditional malaria surveillance. In a traditional malaria surveillance system, data movement is unidirectional, and outputs do not inform community-level response. Additional data are not incorporated into a central database. (**B**) Ideal malaria surveillance. In an ideal malaria surveillance system, all levels contribute data to a central database, the central database provides data analysis and guidelines to all levels, and communication is bi-directional.

Surveillance for malaria control aims to estimate the burden of malaria and inform population-level programs, whereas surveillance for malaria elimination strives to capture and respond to every malaria case.^7^ An ideal malaria elimination information system to support surveillance and response activities collects and transmits data about cases and program activities swiftly, incorporates data from other existing surveillance systems in real time and analyzes data to inform rapid response strategies (Figure 2).^8^

To achieve these aims, the system requires several key features as follows:

### Rapid and complete reporting.

Data should be collected from the lowest level and in the most direct manner possible.^9^ This includes data collected passively at health facilities and in communities from community health workers as well as through active case detection during case investigations or screening activities and intervention data from district-level malaria programs. Consensus on key indicators, or minimum essential data, that a malaria elimination information system needs to capture is fundamental. Complete, timely reporting is an essential element of a malaria elimination surveillance system. Methods for capturing data must be rapid, locally appropriate, feasible, and sustainable by the malaria program. Malaria should be made a notifiable disease once incidence is low enough that malaria surveillance teams can investigate and report every individual case. Instituting a “zero reporting” policy that requires all reporting sites to communicate the number of cases tested and detected regularly, regardless of whether a new case has been detected. This policy, as outlined in the WHO's surveillance guidelines for poliomyelitis and Japanese encephalitis, will further improve data quality.^10^ Zero reporting reduces missing data and helps identify locations where reporting is irregular or incomplete.^11^

### Incorporation of additional data.

Incorporating data sources external to the malaria program, and even the health system, into the malaria elimination information system will improve decision making. For example, the use of census data can provide population denominators, climate and land use data warn of potential areas at risk of outbreaks, and population movement information may indicate the need for a rapid shift in resource targeting. The malaria elimination information system must be flexible enough to receive information from and export to external databases to ensure data can be used by and shared among multiple stakeholders.

### Accessible data storage and management.

Data should be accessible to key members of the health system from the central level down to the implementation units in the communities.^9^,^12^,^13^ The database should be appropriate for local conditions, taking into account existing community-level assets and skills, as well as access to power and equipment repair.^14^,^15^ In some locations, a cloud-based database will make the most sense because anyone with Internet access and administrative clearance can submit and obtain data in real time. However, in locations where reliable Internet access is unavailable, local databases that feed into a central server may help ensure that work can continue during Internet outages. Regardless of the type of data storage used, guidelines for timeliness of reporting must be established.^6^,^16^ Data storage and management systems must be computer based and should include a plan for ongoing maintenance.^9^

### Automated and expert analysis.

A malaria elimination information system should include automated data analysis to ensure timely outputs and expert analysis for policy and programming decisions.^9^,^17^ Automation is vital because a malaria elimination information system must receive and analyze data and output results quickly to identify threats such as outbreaks, inform responses, and monitor the functioning of the whole elimination program. It is essential to incorporate technical assistance and capacity building for malaria program staff at all levels to ensure the database is used effectively. Malaria experts working where the surveillance system is implemented, including in the field, should participate in analysis and interpretation of outputs to ensure that recommended interventions are feasible and reflect local conditions.

Expert analysis can be used to model the expected impact of different combinations of interventions and has been used in other contexts to improve complex processes and systems and decision making.^18^ The models can use data from the surveillance system to help determine which interventions should be used and in what manner to ensure the most impactful, efficient, and cost-effective response.^19^,^20^ The impact of the response can be captured by the surveillance system and can inform further iterative changes to the interventions. Geospatial modeling was conducted in Haiti to produce malaria risk maps as part of an assessment of the feasibility of malaria elimination.^21^ Because of the unreliability of passive surveillance data, parasite prevalence data were used to better understand the temporal and spatial distribution of malaria. From this work, optimal interventions and treatment strategies for various populations and locations were suggested.

### Customized output and feedback.

An ideal malaria information system should automatically generate outputs tailored to the level of the health system that receives them, including visualizations of analyzed data, work task lists, and reports for internal use, external organizations, and donors. Once data are analyzed, visualizing results is essential to effectively share the information. Outputs to the lowest levels should be understandable and directly useful for operational responses, for example, including information that directs the surveillance officer to the household or health facility of the case. Monitoring and evaluation of the outputs are necessary to measure the value added of the malaria elimination information system itself and understand how the system can be improved.

### Targeted response.

Response to malaria elimination information system outputs needs to be timely, effective, and targeted.^9^,^13^,^22^ At the local level, once health staff receive outputs, including a work task list, they must take immediate action.^23^,^24^ This list may include households to be screened for infection, receive preventive interventions such as indoor residual spraying with insecticide, and receive health education. As the workers are implementing their task list, they can also collect data that should be uploaded into the information system that will further inform the intervention strategy, such as coverage and use of interventions, and the occupational risk factors of the people within the target area. The uploading of data from the response activity acts to inform the information system that the activity has taken place operating as a tool for monitoring and evaluation. Findings from the review and key informant interviews highlighted that connecting outputs from the information system to action is the weakest element of existing systems.

## Description of existing Information systems

Currently, few malaria information systems exist that can collect, store, analyze, and provide feedback to implementers based on real-time information. Many existing systems are limited in geographic coverage, do not collect sufficient data to inform rapid response, or are not connected to decision making. While no existing malaria information system contains all the elements listed above, below are examples of existing systems that contain elements of what an ideal system might look like and offer valuable lessons on how to conduct surveillance that can lead to effective responses. Comparisons of these and other systems are highlighted in Table 1.

### China.

The strength of the Chinese Information System for Disease Control and Prevention is its timeliness, ease of reporting, and intuitive 1-3-7 monitoring framework. The 1-3-7 framework dictates that malaria cases be reported within one day, case investigation must occur within three days, and foci investigation and increased prevention measures implemented within 7 days.^6^ The recommended responses vary by the levels of endemicity and risk, with “active and passive surveillance, with particular attention to mobile populations,” in areas with higher incidence, “passive surveillance in the transmission season and active surveillance targeting transmission foci” in zones with seasonal malaria, and “intensified surveillance and response” in border areas.^34^

### Solomon Islands and Vanuatu.

Automated analyses and customized outputs, as well as the potential to guide targeted, rapid response, are the strengths of the Spatial Decision Support System (SDSS), implemented in Vanuatu and the Solomon Islands.^35^ This GIS uses the time and place of malaria cases and intervention coverage to automatically classify areas according to risk and then generate specific response recommendations. The information system creates automated maps of households, including coverage, incidence, and additional geographic and entomologic data. Work task lists are generated for intervention and case management teams for each geo-located house they should visit.

### Swaziland.

The strengths of Swaziland's malaria information system include rapid case reporting through the Immediate Disease Notification System (IDNS), a surveillance system integrated with the reportable disease system, and surveillance outputs that are rapidly relayed to a team that can initiate a response.^36^ The health facility staff members call a toll-free number to report cases to the IDNS, which then sends multiple short message service (SMS) messages with case details to the local malaria program manager and the surveillance team, who investigate within 48 hours. Weekly goals and feedback are provided to surveillance officers to improve coverage and speed of follow-up and screening.

### Zanzibar.

The strengths of Zanzibar's Malaria Case Notification (MCN) system are its rapid reporting and outputs detailing geo-location of cases.^37^ Through MCN, cases are reported in real time and then a tablet-based platform alerts district malaria officers to follow-up, guiding which households are visited to conduct reactive case detection. In this system, surveillance is an intervention, where mobile reporting allows the collection of data in real time that are used to guide a local response.^15^,^38^

## Linkage between regional and global information systems

Ideally, national malaria control and elimination information systems would link seamlessly with related regional and global structures, prioritizing cross-border intelligence sharing information regarding transmission hotspots, outbreaks, and human movement. This would lead to appropriate allocation of national and regional resources and timelier targeted action. However, database linkage between countries and within regions is difficult due to the sensitivity of sharing and nonstandardized collection of data. As more countries move toward malaria elimination and cross-border and regional malaria elimination initiatives are implemented, sharing of data should become a priority. In an effort to facilitate data sharing for malaria control, WHO now coordinates a “situation room” that is focused on the 10 African countries with the highest malaria burden, bringing country representatives together virtually every 2 weeks to discuss stock control, funding issues, and to track current and potential outbreaks. Similar regional situation rooms such as the data sharing hub being developed by the Emergency Response to Artemisinin Resistance in the Greater Mekong Subregion could facilitate data sharing and coordination among malaria-eliminating countries.

## Recommendations

To build a robust and action-oriented malaria elimination information system, a number of key issues require consideration.

### Reporting.

In countries pursuing malaria elimination, when incidence is low enough, rapid reporting of cases should be implemented. Once in the malaria elimination phase reporting must be required by law, preferably within a defined period, and appropriately incentivized in all sectors caring for malaria patients, including private sector health providers and militaries. Reporting should include negative diagnostic test results and zero case reporting.

### Database management.

The malaria elimination database must be manageable by the National Malaria Control Program (NMCP). A malaria elimination surveillance system must provide a framework to guide strategic decision making and support the effective management, coordination, and implementation of interventions. All levels of the malaria control program, from the community to the national level, should receive information from the system. Expert epidemiological and information technology oversight of the system is crucial requiring human resources to support data analysis, including surveillance database managers and epidemiologists who can program database queries, analyze, and interpret data.

### Information and results feedback.

An effective information system must feed analyzed data back to those executing the malaria program, particularly at the community level. In this review, we found few examples of systems that rapidly shared analyzed surveillance information, which could contribute to more rapid and complete responses. For surveillance to function as an intervention, real-time feedback and effective responses are essential. Global stakeholders must take note of this weakness and target investments to improve appropriate rapid feedback from malaria information systems that lead to effective responses.

### Technology.

Locally appropriate technologies, such as mobile phones and web-based systems, can help support data quality improvements and reporting timeliness. Most importantly, comprehensive spatial decision support systems that incorporate GIS are invaluable, as they enable mapping of cases and interventions, automated foci identification, and targeted responses.^35^,^39^

### Data sharing and commitment.

Real-time sharing of standardized malaria data across borders has the potential to contribute to malaria elimination. A key element of malaria elimination programs is rapid and appropriate response to malaria cases. Standardized and streamlined methods and indicators will improve reporting and decision making. Interventions will need to be adapted to the location and population; however, there is an urgent need for generic and adaptable standard operating procedures on which NMCPs can base their surveillance and response strategies. The effective implementation of regional collaborations within malarious regions looking to eliminate may be crucial for the success of national and regional malaria elimination. Currently there are few functioning cross-border malaria elimination collaborations. Ideally, surveillance systems would be unified across countries and would incorporate information from militaries who liaise with government and civilian authorities. Harmonizing existing surveillance systems will require both political and financial commitments in short term and long term. In short-term, commitment is needed to bring stakeholders together to develop political and financial capital for malaria elimination surveillance and information systems. Malaria elimination is a long-term strategy, therefore, commitment is needed to maintain a cadre of workers who can work with the software and adapt it to fit changing circumstances.

Many new technology developments to improve surveillance for malaria elimination appear attractive for investment. However, an investment in technologies is not a panacea; a surveillance system is only as good as its implementation. An excellent information system should be at the core of malaria elimination programs to ensure that all cases are detected and responded to in an effective and timely manner. Investment in robust, response-focused systems is essential to achieve malaria elimination.

## Figures and Tables

**Table 1:** Existing surveillance systems for malaria elimination

Country	System description	Data capture	Outputs	Strengths	Challenges
Cambodia	MIS is a stand-alone system developed to assess malaria transmission and intervention coverage.^25^ Two additional pilot systems	Passive Case Detection case notification	MIS: Automatically generated report including tabular summaries, graphics and mapping to village level^26^	MIS:	Uncaptured private, migrant, military sectors
	D0AS P*f* cases	MIS: District level data reported monthly, including species, severe malaria, cases, deaths	D0AS: Real-time SMS alert to Provincial Health Department and National Malaria Center. Day-28 follow-up reminder is sent to the same plus health center management	Covers all endemic areas	Most data aggregated monthly, challenge to get real-time data
	D3AS Day 3 positive malaria smears to identify resistance	D0AS: Health staff send SMS for *Pf* cases from pilot areas	D3AS: Real-time SMS when parasites remain by Day 3	Tracks severe malaria, deaths	Inconsistent decision making and response based on available data
	Population covered: > 3M	D3AS: Only includes *Pf* cases parasitemic after three days of treatment in areas of artemisinin-resistance		Malaria incidence and intervention coverage to village level	Does not capture time-to-case reporting, or intervention quality
				Automatically generated monthly bulletin	Case follow-up challenges
				Pilot D0AS and D3AS	No mapping to household or where case acquired
				SMS and Internet-based notification systems	
				Integrated with MIS	
China	Two integrated web-based systems: febrile illness reporting and focus investigation and intervention tracking. Data stored at the National Centers for Disease Control and Prevention.^6^,^27^	PCD case notification: Data entered within 24 hours. Data include date, facility, reporting person, patient info and diagnostic result with method and treatment	SMS alerts	Web-based system integrated with reportable diseases system	Mobile technology not integrated
	Population covered: > 1.3B		Monthly MoH report, tabular summary results, graphics and mapping	Data fed into HMIS	Limited baseline data
			“1-3-7 strategy” time tracking to case notification (one day), case investigation (three days), completed interventions (seven days)	Very little missing data	Does not capture new interventions or intervention quality
				Rapid case reporting	No mapping to household or where case acquired
				Diagnosis is confirmed by microscopy and PCR	
				“1-3-7 strategy” is easy to use and understand	
Solomon Islands/Vanuatu	SDSS.^17^,^35^ Data are stored in a relational database, using local, provincial and nationally based servers (three levels for backup).	PCD case notification: Health facility calls provincial center within 48 hours.	Real-time case reporting	SDSS includes extensive baseline data^28^	Mobile technology not integrated
	Population covered: > 90 k, implemented in four island provinces		Frontline and active case detection planning by household, follow-up list of households that did not receive intervention	Rapid case reporting	Inconsistent decision making and response
			Tabular output, spatial analysis, graphics, and mapping, including foci classification	Automated GIS-based queries with high-resolution mapping	Does not capture time-to-case reporting, or intervention quality
				Generates lists to support targeted action at the household level	Human resource constraints
				Readily adaptable to other locations or systems	No mapping to where case was acquired
Swaziland	HMIS, IDNS for 15 reportable diseases, and MSDS for case investigation and interventions.^16^,^29^,^36^	PCD case notification: RDT or microscopy-confirmed malaria cases dictated through a toll-free hotline. Data entered on a central server, surveillance agent receives an SMS with date, facility, reporting person, patient info and case number to conduct case investigation and intervention.	IDNS: Toll-free hotline resulting in SMS to surveillance agent	Integrated with notifiable disease reporting system	Relatively low reporting completeness to IDNS
	Population covered: 1.2M		MSDS: Monthly tabular and graphic summary, mapping to household. Maps of cases investigated, locations of positive cases, IRS, ITNs, breeding sites, risk maps, households screened, or remaining.	Web-based system using mobile technology	Low case reporting from private sector facilities
				Free mobile reporting	Does not capture time-to-case reporting or intervention quality
				Entire country covered	No mapping to where case was acquired
				Simple, rapid case notification	
				Temporal–spatial analysis of case distribution	
Thailand	Stand-alone, web-based system. Data storage is in a database at Mahidol University. GPS-enabled tablets for patient follow-up, data captured in same server.	PCD case notification: Case data entered at malaria clinic level within 24 hours. Data include date, facility, reporting person, patient info, diagnostic result with method and foci classification.^30^	*Pf* case alerts to malaria clinic staffs' tablets within 24 hours	Web-based system with mobile technology being integrated	Hospital-based cases in a separate system
	Population covered: > 21M		Tablet-based follow-up form for directly observed therapy and resistance monitoring	Implemented in large regions, covering all areas of multi-drug resistance	Challenges with migrant and cross-border follow-up
			Monthly MoH report, tabular summary, graphics, maps, with mapping to *Pf* case household and likely case location.^31^	Rapid case reporting	More baseline data needed, such as intervention coverage and forest sleeping locations
				Captures DOT	No time to case reporting or intervention quality
				Captures *Pf* resistance	
Zambia	DHIS2 is a web-based health information system. Data storage and mobile phones linked to the same database.^32^,^33^	PCD case notification: urban and rural health staff report weekly by mobile phone. Data include clinic visits, clinical cases, RDT-tested and positive cases, microscopy-tested and positive cases, ACT and RDT stock tracking. CHWs report cases monthly by mobile phone.^33^	Regular reports, with online access to data in real-time	Open source free web-based system fully integrated with HMIS	Case data not reported to DHIS2 in real-time
	Population covered: > 6M		Graphs created and provided in real time to mobile phones or computers, summarizing case reporting and stock data, with summary data from all areas, reporting to the facility	Tables, charts and maps shared with all users with online dashboard	Does not capture time to case reporting or intervention quality
			Maps, graphs display village, clinic-level malaria incidence	Mobile technology fully integrated	Remains to be determined if DHIS2 can support full malaria elimination surveillance system to household level
				Timeliness and completeness of data reporting tracked	
Zanzibar, Tanzania	Integrated system combining Coconut Surveillance and MCN. MCN includes rapid reporting and analysis, outputs with geo-location of cases, through Coconut Surveillance. Cases reported to health staff via SMS. Coconut uses data to guide household oriented index case follow up.	PCD case notification:	MCN: Real-time case reporting via Coconut Surveillance, monthly MoH reports. Tabular summary results, graphics and mapping to the village level.	MCN and Coconut are an integrated SMS-based system and tablet web-based system	Cases from extensive private sector not captured
	Population covered: ∼1.3M	Public health unit staff send an SMS for each positive case. Data include all-cause visits, malaria tested/positive cases and age.	Coconut: Real-time tabular summary results, graphics, and detailed mapping to the household level. Real-time tracking of case follow-up and new interventions.	Mobile technology fully integrated	Limited capture of baseline data
		Coconut Surveillance notifies malaria officers of cases immediately via SMS. Patient and household follow-up with GPS enabled tablet.		Rapid case reporting	Does not currently capture intervention quality
				Real-time tabular output of key variables makes it easy for management to track progress real time	No mapping to where case was acquired
				MEEDS data are used to calculate supply orders	Denominator (population) data not captured with Coconut

D0AS = Day 0 Alert System; D3AS = Day 3 Alert System; DOT = directly observed therapy; HMIS = Health Management Information System; GIS = geographic information systems: MCN = malaria case notification; IDNS = Immediate Disease Notification System; MIS = Malaria Information System; MoH = Ministry of Health; MSDS = Malaria Surveillance Database System; P*f* = *Plasmodium falciparum*; PCR = polymerase chain reaction; SDSS = Integrated Spatial Decision Support System; SMS = short message service.
